# Health professionals’ use of smartphone apps for clients with low back pain: an observational study

**DOI:** 10.1017/S1463423625000209

**Published:** 2025-02-24

**Authors:** Claudia Didyk, Lucy Kate Lewis, Belinda Lange

**Affiliations:** Caring Futures Institute, College of Nursing and Health Sciences, Flinders University, Adelaide, Australia

**Keywords:** Barriers, behaviour change, health professional, low back pain, self-management, smartphone apps

## Abstract

**Aim:**

This study aimed to explore health professionals’ use, barriers, confidence, and preferences for technology and smartphone apps to assist clients with self-managing low back pain (LBP).

**Methods:**

Prospective observational cross-sectional survey of registered Australian health professionals that managed clients with LBP.

**Results:**

In total, 52 survey responses were included (mean age 43 ±13.8 years). Most did not personally use healthy lifestyle apps (60%) and did not recommend apps due to a lack of knowledge of app effectiveness (93%). The largest barrier to recommending apps was the potential for apps to be misused as a substitute to health professional diagnosis. Fifteen recommended smartphone apps (mean age 36 ±10.6 years) and were at least moderately confident in choosing/recommending apps (94%) and assessing app quality (80%). Those more likely to recommend apps personally used apps for healthy lifestyle behaviours (odds ratio (OR) 5.1 (p = 0.009)) were physiotherapists (OR 0.13 (p = 0.035) c/f chiropractors in their profession for <10 years (OR 8.6 (p = 0.015)) c/f >30 years. Increasing age decreased the odds (OR 0.94 (p = 0.013)) of recommending apps.

**Conclusions:**

Health professionals do not recommend LBP self-management apps due to a lack of knowledge of their effectiveness. Those that do recommend apps are confident with app choice, recommendation, and app quality assessment. Physiotherapists with <10 years’ experience were most likely to recommend apps.

## Introduction

Prior to 2020, the use of digital tools in health management and mobile app use was declining globally, (Accenture, 2020). The COVID-19 pandemic and subsequent restrictions caused increased reliance on digital health options for consumers and health professionals, due to both the lack of face-to-face options and an increase in availability of modalities such as telehealth. This shift towards broader acceptance and access to digital health options for consumers and health professionals has shown no sign of slowing down, with digital options now integrated into sustainable models of health care. There are opportunities for increased adoption of a broader scope of digital health options such as smartphone applications (apps) that may assist in the management of conditions that are a global burden, such as low back pain (LBP) (Alene *et al.*, 2024, Briggs *et al.*, 2021, Chhabra *et al.*, 2023).

LBP is the leading cause of disability globally, the most prevalent condition requiring rehabilitation during the course of the injury (568 million people), and poses a significant economic challenge (Cieza *et al.*, 2020, Vos *et al.*, 2017). Digital tools, such as smartphone apps, offer an easily accessible and cost-effective option for scalable health management (Deniz-Garcia *et al.*, 2023). Smartphones are ubiquitous, and apps have the potential to address health inequities in low- to middle-income countries, rural and remote communities (Deniz-Garcia *et al.*, 2023). While smartphone apps can be used as a useful adjunct to face to face management (Wang *et al.*, 2018), recent data show that uptake for musculoskeletal conditions that make up a large component of visits to health professionals is low (Gordon *et al.*, 2020, Gupta *et al.*, 2023). Musculoskeletal conditions make up 18 per cent of visits to general practitioners in Australia (Australian Institute of Health and Welfare [AIHW], 2022). With the current shortage of general practitioners (Australian Medical Association [AMA], 2022), timely access to medical care can be impeded by appointment utilization for musculoskeletal complaints (AMA, 2022). In Australia, back problems account for nearly a quarter of health system expenditure (AIHW, 2022), resulting in unnecessary presentations to hospitals, with people with LBP occupying hospital beds required for medical clients (Coombs *et al.*, 2021), and this trend is similar in other countries (Beyera *et al.*, 2020, Edwards *et al.*, 2017, Melman *et al.*, 2023). Current clinical practice has been explored by internationals studies to increase LBP self-management (Adam *et al.*, 2020; Banerjee *et al.*, 2022; Chala *et al.*, 2022; Kongsted *et al.*, 2021). Change in the management of LBP is required, with a need to move to increased self-management and consumer independence in their own care.

The National Institute for Health and Care Excellence LBP guideline (NICE, 2016) recommends self-management and exercise as the gold standard for LBP management. The self-management recommendations include advice and information tailored to the person’s needs and capabilities; encouragement to continue with normal activities and active rehabilitation such as exercise. The rehabilitation should consider the individual’s needs, preferences, and capabilities. Exercise could include biomechanical, mind-body or a combination as well as group exercise in conjunction with psychological therapies using a cognitive behavioural approach. Finally, programmes are recommended that facilitate return to work and normal activities. Digital health management tools, such as smartphone apps, may offer an easily accessible, cost-effective, guideline informed (NICE, 2016) LBP self-management solution that may improve self-efficacy and encourage consumer independence in their care.

Although apps can be used directly by consumers, consumers look to health professionals for guidance (Accenture, 2020; Byambasuren *et al.*, 2020) and recommendation of apps (Byambasuren *et al.*, 2020) to facilitate appropriate app choice, which requires health professionals to have the knowledge, and tools, to do so. Recent data show that over half of consumers from Australia, England, Finland, Norway, Singapore, Spain, and the United States, who have used digital health care are willing to receive it from their usual healthcare providers; however, only 11 per cent of health care providers recommend digital tools for health management (Accenture, 2020). This mismatch may be due to data protection concerns, time constraints, and medical liability, which are identified barriers of in-clinic use of digital health management tools, such as smartphone apps (Sarradon-Eck *et al.*, 2021). There is a clear need to explore and better understand smartphone LBP self-management app use by first point of contact health professionals for LBP management. This information may be applied to address the barriers to adoption of smartphone LBP self-management app use in clinical practice which could be applicable across different countries and health systems.

Therefore, the aim of this study was to explore first point of contact health professionals’ use, barriers, confidence, and preferences for technology and smartphone apps to assist clients with self-managing LBP. The secondary aim was to describe the characteristics of health professionals who use smartphone apps for client management of LBP, including app preferences.

## Methods

### Design

This prospective observational cross-sectional online survey (Qualtrics) explored the use, barriers, confidence, and preferences for technology and smartphone apps for people with LBP from a health professional perspective. Ethical approval was gained from the Flinders University Human Research Ethics Committee (HREC) (no. 2818). Prior to undertaking the survey, online informed consent was gained from all participants.

### Participants

Participants were health professionals who were considered first point of contact for LBP management (Chinese medicine practitioners, chiropractors, exercise physiologists, general practitioners, occupational therapists, osteopaths, physiotherapists, podiatrists, and psychologists) (AIHW, Arthritis Australia: My Back Pain, 2016), registered in Australia and either currently or had previously managed clients with LBP of any duration. Health professionals were recruited, from April to July 2022, through relevant national health professional associations (e.g., Australian Physiotherapy Association) (via email with a link to the Qualtrics survey and a reminder email at two weeks if required), Facebook paid posts on a designated study, non-personal page, and via the Flinders University webpage where volunteers can select to participate in research studies and surveys (via an anonymous link to the Qualtrics survey). A purposive sampling method was used to intentionally select participants based on their health profession and management of clients with LBP. Pilot testing participants were recruited via email to personal networks of the research team with a link to the to the Qualtrics survey. The pilot testing survey contained an additional box for feedback.

### Survey instrument

A comprehensive literature review did not identify existing validated surveys or instruments that measured health professionals’ use of smartphone apps for client management of LBP. The research team developed a survey, incorporating pre-existing instruments relating to the self-management of LBP.

The final survey consisted of 27 items (24 closed, 3 open-ended) in five sections:

- *Personal information* was collected including age, gender, highest level of education, current profession and years of experience, work setting and location, and access to technology.
- *Smartphone app use and preferences –* The survey included a question relating to smartphone app use to help clients self-manage LBP. For those that indicated that they did not use apps, a follow-up question regarding *barriers to app* use was included (Sarradon-Eck *et al.*, 2021). For health professionals who indicated they used apps to assist clients with self-management of their LBP, follow-up questions were included about their *app preferences* in terms of the features that they liked or disliked, relating to *app quality* and the potential of the apps for development of client *self-management and behaviour change.* The survey included a question relating to the personal use of apps by health professionals to promote healthy lifestyle behaviours. This question allowed us to explore the connection between personal app use and experience in choosing and recommending apps. Additionally, previous literature has shown that the personal health behaviours of health professionals may influence their clinical practice (While, 2015; Fei *et al.*, 2013) and that health professionals who model healthy lifestyle behaviours are perceived as more motivating and credible when promoting healthy behaviours (Leske *et al.*, 2012).
- *Self-management potential –* The items relating to the health professionals’ perception of the self-management potential features of apps were developed using the Self-management Support Checklist (SMS-14), developed from the well-established Stanford Self-management Support Model (Devan *et al.*, 2019). The checklist guided the development of survey questions using the six self-management skill categories (1) self-efficacy building, (2) self-tailoring, (3) self-monitoring of symptoms, (4) goal setting and planning, (5) problem solving, and (6) partnership between views of patient and clinicians.
- *Behaviour change potential –*The items relating to the health professionals’ perception of the behaviour change potential features of apps were developed using the App Behaviour Change Scale (ABACUS), a reliable tool with high interrater reliability and internal consistency, evaluating four broad categories of behaviour change (knowledge and information, goals and planning, feedback and monitoring, and actions) (McKay *et al.*, 2019). The Capability, Opportunity, and Motivation Behaviour system model (COM-B) (Michie *et al.*, 2011) is considered a key theoretical framework for supporting behaviour change (NICE, 2014) and was also used to guide survey question development. Questions incorporated the COM-B relating to education, persuasion, incentivization, coercion, training, restriction, environmental restructuring, modelling, and enablement required in behaviour change interventions (Michie *et al.*, 2011).
- *App quality –* Health professional perception of the *quality of the apps* they used was assessed using the Mobile App Rating Scale (MARS), a reliable tool with excellent interrater reliability and internal consistency, evaluating four quality categories (engagement, functionality, aesthetics, and information) (Stoyanov *et al.*, 2015) to guide question development.

The survey was pilot tested for usability (time taken to complete, flow of questions, and clarity) on a group of adults (n = 4, occupational therapist, physiotherapist, chiropractor, and general practitioner). The survey was revised based on this feedback.

### Data management and analysis

Data were exported into Microsoft Excel. Survey responses that included minimal information, only demographic details or were repeated bot responses, were considered invalid and removed. Bot responses were identified through patterns and inconsistencies in responses. Once identified, the possible bot responses were reviewed and discussed by the research team to ensure agreement. The included survey responses with questions in which respondents answered the first component of a question and then did not answer the next component were treated as missing data. Missing data were omitted, and complete case analysis was used. The final complete dataset was exported into the Statistical Package for Social Science (IBM SPSS) version 28.0.1.1 for Windows for analysis. Descriptive analyses were completed on all variables. Associations between health professional characteristics, technology use, and recommendation of smartphone apps were explored using logistic regression analyses. Significance was set at 0.05.

## Results

A total of 100 participants completed the survey, with 52 complete survey responses included in the final analyses.

### Participant characteristics and app use

#### Whole sample

Participants (n = 52) had a mean age of 42.8 (±13.8) years (range 21 to 70 years) and 56% were male (Table 1). Most participants were chiropractors (28%, n = 14) and physiotherapists (24%, n = 12), had 0–9 years (44%, n = 23), or over 30 years (27%, n = 14) of experience, worked in a metropolitan area (56%, n = 29), community (primary care, private practice) (75%, n = 39) setting, with a highest education level of Masters or Doctoral degree (48%, n = 25). Of the 52 participants, 10 (19%) were aware of the NICE LBP guidelines, and one participant correctly outlined all the guideline recommended areas of self-management with the exception of return-to-work facilitation.

**Table 1.** Participant characteristics of whole sample and health professionals that recommend apps for LBP

	Whole sample (n = 52)	Recommend apps (n = 19)
**Gender n (%)**		
Female	21 (40.4%)	5 (26.3 %)
Male	29 (55.8%)	14 (73.7%)
Prefer not to say	2 (3.8%)	–
**Age** (years) mean ± SD (range)	42.8 ± 13.82 (21–70)	35.67 ± 9.71 (21–60)
**Highest education n (%)**		
High school completed	1 (1.9%)	1 (5.3%)
TAFE or trade school	3 (5.8%)	2 (10.5%)
Bachelor degree	15 (28.8%)	7 (36.8%)
Graduate certificate or diploma	8 (15.4%)	4 (21.2%)
Masters or doctoral degree	25 (48.1%)	5 (26.3%)
**Occupation n (%)**		
Chiropractors Physiotherapists Eastern medicine – TCM/Acupuncture General practitioners Exercise physiology/OccupationalRehabilitation Psychologist/Mental health Osteopaths Massage/Myotherapist Did not specify	14 (28.0%) 12 (24.0%) 8 (16.0%) 6 (12.0%) 4 (8.0%) 3 (6.0%) 2 (4.0%) 1 (2.0%) 2 (4.0%)	2 (10.5%) 7 (36.8%) 3 (15.8%) 5 (26.3%) 2 (10.5%) − − − −
**Years in current profession n (%)**		
0–9 years 10–19 years 20–29 years over 30 years	23 (44.2%) 11 (21.2%) 4 (7.7%) 14 (26.9%)	14 (73.7%) 2 (10.5%) 1 (5.3%) 2 (10.5%)
**Work setting n (%)**		
Community (primary care, private practice) Hospital Combination of hospital and community also worked in other settings: Community (not primary care or private practice) Education Sporting team	39 (75%) 6 (11.5%) 7 (13.5%) 6 (11.5%) 3 (5.8%) 2 (3.8%) 1 (1.9%)	11 (57.9%) 4 (21.1%) 4 (21.1%) − − − −
**Work location n (%)**		
Metro Non-metro Combination metro/non-metro	29 (55.8%) 15 (28.8%) 8 (15.4%)	11 (57.9%) 6 (31.6%) 2 (10.5%)

Highest education – additional training outside TAFE or trade school was not offered as an option.

Most participants (76%, n = 40) had access to an iPhone (67%, n = 35) or Android phone (48%, n = 25) and 75% (n = 39) had access to a tablet device. One participant did not have access to any smartphone or tablet device. Over 39% (n = 20) of participants reported personally using apps to promote healthy lifestyle behaviours. Technology for client-related care was used by 60% (n = 31) of participants. Thirty-seven per cent (n = 19) of participants recommended apps to clients and 31% (n = 16) reported that clients requested them to recommend LBP apps.

#### Health professionals who recommend apps

Of the 19 participants who reported recommending apps to clients, 15 completed follow-up survey items relating to apps (Table 1). Sixty per cent (n = 9) recommended apps to clients on a weekly basis, mostly for self-management (100%, n = 15), health education (87%, n = 13), and health promotion (87%, n = 13). Health professionals who reported recommending apps to clients were aged 35.7 years (±9.7, range 21 to 60 years) and predominantly male (74%, n = 14). The highest proportion of health professionals using apps for clients were physiotherapists (37%, n = 7), followed by general practitioners (26%, n = 5), had 0–9 years (74%, n = 14) of experience, and worked in a metropolitan (58%, n = 11), community (primary care, private practice) (58%, n = 11) setting.

Ten health professionals (67%) who used apps for clients also reported personally using apps to promote a healthy lifestyle. Most health professionals who recommended apps reported moderate to high confidence in choosing and recommending apps (93%, n = 14) and assessing LBP app quality (79%, n = 11).

### Barriers and facilitators to health professionals recommending apps

For the health professionals who did not recommend apps for clients with LBP (n = 36), the most common reason cited was a lack of knowledge of app effectiveness (93%, n = 27), followed by lack of perceived client digital technology literacy (79%, n = 23).

For the health professionals who recommended apps and responded to follow-up questions (n= 13), 77% (n = 10) reported barriers to recommending apps including: the potential for apps to be misused as a substitute to health professional diagnosis, too time consuming, not independently certified, clinically valid or not backed by evidence, and the potential for medical liability. Data protection concerns or not being sure of what value apps would add to improve outcomes were not considered barriers to app recommendation by 46% (n = 6) of participants. Nine participants suggested that additional elements such as ease of use of apps, better control and customization options, more targeted and/or specific content, and additional education in self-management would encourage health professional recommendation of apps for clients with LBP.

### Health professionals’ preferences for apps for the self-management of LBP

Fifteen health professionals reported recommending a range of apps to clients for their LBP, including: Curable (Curable Inc), Tapping solution (The Tapping Solution, LLC), Keep (Google, LLC), Physitrack (Physitrack PLC), Insight timer (Insight Network Inc), SelfBack (SelfBack), Kaia (Kaia Health), and activity monitors (specific apps not mentioned).

**Figure 1.**
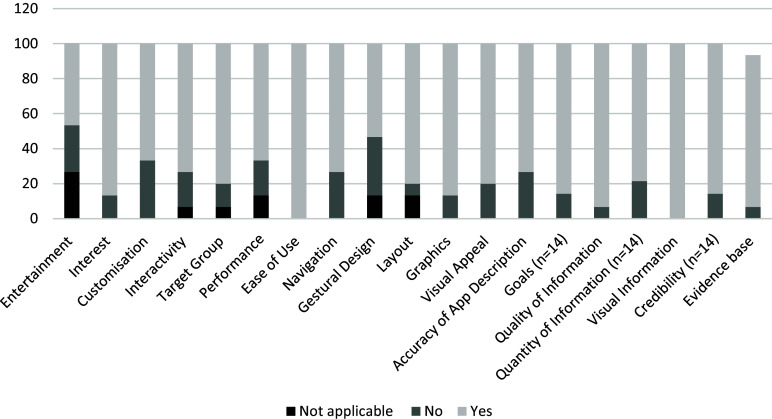
LBP self-management app features liked by health professionals.

Features included and reported as most liked by participants in the apps they recommended (Figure 1) were in the following categories: ‘information’ (visual information (100%, n = 15), quality of information (93 %, n = 14) and evidence base (93%, n = 13)) and ‘functionality’ (ease of use (100%, n = 15)). Features that were disliked by participants were in the ‘functionality’ (gestural design (39%, n = 5)) category as well as the ‘engagement’ (entertainment (36%, n = 4) and customization (33%, n = 5)) category. The features that were most often not included in their chosen apps were in the categories of ‘engagement’ (entertainment (25%, n = 4)) and ‘functionality’ (performance (19%, n = 3)).

**Figure 2.**
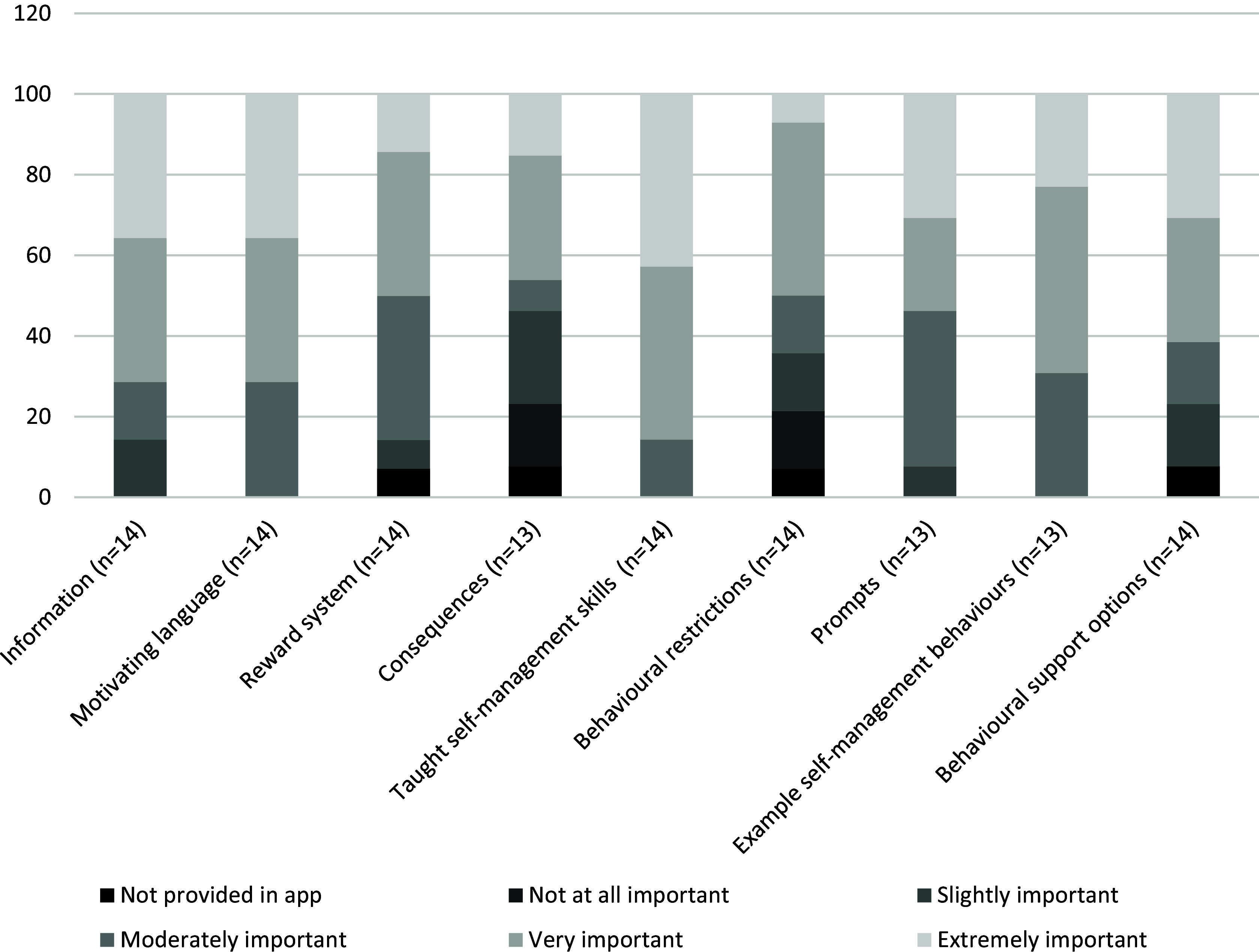
Behaviour change features and perceived importance of apps recommended by health professionals.

Features considered by health professionals when investigating LBP self-management apps included goal setting (100%, n = 15), prompts and alarms (93%, n = 14), advice (93%, n = 13), education (87%, n = 13), monitoring (87%, n=13), planning (88%, n = 12), and social support (80%, n = 12). The least sought-after feature was personalization (73%, n = 11). For the apps that had these features, health professionals rated the following as very or extremely important for LBP self-management apps: teaching skills required to self-manage (86%, n = 12), providing information to promote self-management (71%, n = 10), using motivating language to encourage self-management (71%, n = 10), providing examples of self-management behaviours to follow (69%, n = 9), and prompts to encourage self-management (54%, n = 7) (Figure 2). A small number of participants considered consequences for not following self-management advice (17%, n = 2) and behavioural restrictions (15%, n = 2) not at all important.

### Associations between health professional characteristics, technology use, and recommendation of smartphone apps

Age, current profession, and years of experience were significantly associated with app recommendation. With every year increase in age, the odds of recommending apps was less (OR 0.94; p = 0.013). Chiropractors were less likely to recommend apps than physiotherapists (OR 0.13, p = 0.035), and those in their profession with less than 10 years of experience were more likely to recommend apps (OR 8.6, p = 0.015) than those with over 30 years of experience.

Access to a smartphone was statistically associated with app recommendation, with health professionals with access to an iPhone (OR 1.8, p = 0.008) or a Windows smartphone (OR 4.9, p = 0.012) being more likely to recommend apps. Participants were more likely to recommend apps if they personally used apps to promote their own healthy lifestyle behaviours (OR 5.1, p = 0.009) (Table 2).

**Table 2.** Associations between demographic factors, technology use, and app recommendation

	**App Recommendation** (n = 19) O/R S.E. (p value) [95% CI]
**Gender** (n = 50) (Male)	3.2 0.637 (p = 0.068) [0.919, 11.145]
**Age** (n = 50) (years)	0.9 0.027 **(p = 0.013)** [0.886, 0.986]
**Highest education** (n = 51) (TAFE or below)	5.8 1.195 (p = 0.141) [0.559, 60.475] bachelor or above
**Current profession** (n = 51) (Physiotherapist)	3.6 1.242 (p = 0.305) [ 0.313, 40.751] GP 0.0 28420.7 (p = 0.999) [ 0.000] Osteopath * 0.1 0.966 **(p=0.035)** [0.020, 0.863] Chiropractors 0.3 0.787 (p = 0.101) [ 0.059, 1.285] Other professions
**Years in current profession** (n = 51) (Over 30 years)	8.6 0.879 **(p = 0.015)** [1.526, 47.956] 0–9 years 1.2 1.096 (p = 0.855) [0.143, 10.480] 10–19 years 1.8 1.387 (p = 0.662) [0.121, 27.797] 20–29 years
**Work setting** (n =51) (Community)	4.9 0.937 (p=0.089) [0.782, 30.801] hospital 3.3 0.843 (p=0.160) [0.627, 17.092] combined settings
**Work location** (n = 51) (Metro)	1.2 0.662 (p = 0.757) [0.335, 4.491] non-metro 0.6 0.902 (p = 0.501) [0.093, 3.194] combined locations
**Access to smartphone – Iphone** (Yes)	1.8 1.087 **(p = 0.008)** [2.140, 151.402]
**Access to smartphone – Android** (No)	1.6 0.584 (p = 0.448) [0.496, 4.898]
**Access to smartphone – Windows** (Yes)	4.9 0.631 **(p = 0.012)** [1.424, 16.930]
**Access to Tablet – Ipad** (Yes)	3.1 0.641 (p=0.079) [0.879, 10.829]
**Access to Tablet – Android** (Yes)	2.6 0.631 (p=0.131) [0.753, 8.995]
**Access to Tablet – Windows** (Yes)	3.0 0.601 (p = 0.066) [0.931, 9.827]
**Access to other** (No)	6.9 1.105 (p = 0.081) [0.786, 59.814]
**Personally use apps** (Yes)	5.1 0.627 **(p = 0.009)** [1.506, 17.568]

**BOLD =** significant p = 0.05.

* = None in this sample.

Logistic regression analyses were used for the results.

## Discussion

This study aimed to investigate health professionals’ use, barriers, confidence, and preferences for technology and smartphone apps to assist clients with self-managing LBP. Most Australian health professionals were at least moderately confident in choosing or recommending apps and assessing their quality. Almost one-third of health professionals had clients request recommendation of LBP self-management apps, and over one-third recommended LBP self-management apps to clients. Most health professionals who recommended apps did so on a weekly basis for LBP self-management, health education, and health promotion. Physiotherapists, higher levels of education, increasing age, less than 10 years’ experience, and personally using healthy lifestyle apps were associated with greater app recommendation to clients. A lack of knowledge of app effectiveness and perceived lack of client digital technology literacy were identified barriers to app recommendation.

Interestingly, in this study, we found much higher rates of app recommendation by health professionals, over three times higher than published trends (Accenture, 2020). It is possible that there has been a large increase in the number of health professionals using and recommending digital options to clients in the last three years, aligned with the global move to telehealth and virtual options in developed countries during the COVID-19 pandemic (Webster, 2020, University of Queensland [UQ], 2022, Thomas *et al.*, 2020). In this study, physiotherapists with less than 10 years’ experience were more likely to recommend apps. Incorporating technology into client care may be easier for those newer to the workforce, due to greater technology literacy and an increased likelihood that these professionals were exposed to these technologies during their health professional training (Martin *et al.*, 2022). We also found that approximately one-third of clients requested LBP self-management apps to be recommended by their health professional, suggesting that clients may take the lead from their health professional regarding possible digital options for their LBP self-management.

Clients trust health professional guidance in managing their health (Accenture, 2020, Deloitte, 2018) and guideline-based recommendations are considered best practice to improve outcomes for people with LBP (Foster *et al.*, 2018). Not adhering to these recommendations could result in poorer outcomes and greater personal and health system costs. Despite the high prevalence of LBP and the high levels of health care usage and presentation to health care providers, less than two per cent of health professionals in this study were able to correctly identify most of the NICE (2016) LBP guideline self-management recommendations. This lack of knowledge could be due to health professionals considering guidelines to be restrictive on professional autonomy, clinical reasoning, and patient empowerment (Correa *et al.*, 2020, Peters *et al.*, 2020, Slade *et al.*, 2016, Wang *et al.*, 2023). It is also possible that health professionals are not exposed to these guidelines throughout their entry-level training or ongoing professional development (Derghazarian and Simmonds, 2011, Synnott *et al.*, 2015). This lack of knowledge of best practice guidelines is concerning and warrants further investigation. Of further concern is that some health professionals reported using management options, such as electrotherapies, that are not currently recommended for LBP (NICE, 2016, Zadro *et al.*, 2019). Return to work facilitation was the least identified recommendation, which is surprising as LBP presents mostly in working age and is a leading cause for early retirement in Australia (Schofield *et al.*, 2012). International guideline-based self-management recommendations, particularly in the facilitation of return to work, could improve patient outcomes, allow people to remain at work, and reduce the chance of decreased wealth in later life due to early work cessation. The lack of knowledge of guidelines and use of guideline-based recommendation should be addressed in continuing education for health professionals involved in the management of people with LBP (Hush and Alison, 2011).

Behaviour change techniques can encourage people with LBP to adopt health behaviours to self-manage their condition (Mansell *et al.*, 2016), and LBP self-management interventions may improve health outcomes by including features that encourage behaviour change (Araújo-Soares *et al.*, 2019). In contradiction to current literature (Didyk *et al.*, 2022), this study found that the apps recommended by health professionals included many features to promote behaviour change. However, most of the recommended apps were not specific for LBP self-management and did not address the required behaviour change and self-management support criteria for LBP self-management interventions (Didyk *et al.*, 2022, Michie *et al.*, 2011). Health professionals placed importance on example self-management behaviours and skills, behavioural restrictions, and sought goal setting and prompts and alarms as self-management features. Health professionals also liked features relating to app quality functionality (ease of use), information (visual information, quality of information, evidence base, goals, and credibility), aesthetics (layout, graphics), and engagement (interest, target group). Functionality and aesthetic features have been previously found to rate highly, and although highly liked by health professionals, the information and engagement features were rated the lowest quality in a previous systematic app assessment (Didyk *et al.*, 2022). This could be due to health professionals recommending different apps to those formally assessed in previous research. Simple app assessment methods or tools for health professionals to assess app quality may assist with future recommendation of apps for clients (Gordon *et al.*, 2020).

Recommendation of smartphone apps needs to be part of workflows nested into current health care delivery models (Accenture, 2020). Barriers to implementation of app use into clinical practice, such as lack of time, exist. Sustainable adoption of smartphone app use for the self-management of LBP will need to address health professional time constraints. One of the largest barriers for health professionals recommending apps was the potential for apps to be misused as a substitute to health professional diagnosis. To minimize this, oversight should be provided by trusted health professionals (Bernhardsson *et al.*, 2019). Whilst many commercially available apps in the app stores are not independently certified, clinically valid, or backed by evidence, some guidance from health care providers could ensure better app choices aligned with client needs. Further education on the value of recommending apps to suitable clients to improve outcomes may assist with greater app recommendation (Gordon *et al.*, 2020). Health professional app recommendation would also need to be client specific and take into consideration levels of digital technology literacy (Kloek *et al.*, 2020). Potential for medical liability could be avoided by recommending important features that should be present in apps to assist in self-management and behaviour change. Educating health professionals about app features for self-management and behaviour change potential (Gordon *et al.*, 2020) could increase health professional confidence in app effectiveness and increase use. This could assist both health professionals and consumers to determine suitable LBP self-management apps, reduce liability, and increase confidence. There is a clear need for an app evaluation tool (Gordon *et al.*, 2020) that is quick and easy to use, which may increase LBP self-management app use by people with LBP and recommendation by health professionals.

This study has numerous methodological strengths. Dissemination occurred nationally, using free and paid online methods. Reliable and validated tools were used in the survey for a high level of confidence in survey results. Health professionals can be challenging to recruit due to their time constraints and workforce shortages (AMA, 2022). Data were collected from over 50 health professionals, representing different first point of contact disciplines and of relevance to a range of primary care health professionals. There is little known about the current use of apps in health care, and more specifically for LBP self-management. A large amount of data were collected from over 50 health professional respondents nationally. Although the number of health professionals who recommended apps was small, it was over three times higher than expected from previous literature (Accenture, 2020, Deloitte, 2018). There are also some limitations that must be acknowledged. It is possible that the online recruitment method used in this study was biased towards recruiting health professionals comfortable in an online digital environment and potentially more likely to recommend apps to their clients. Additional training outside TAFE or trade school was not offered as an option. The health professions listed include eastern medicine and massage therapist, both of which may have training outside of TAFE/trade school/University. This may be why one participant selected high school as the highest formal education. Additionally, despite recruitment efforts, the number of health professionals recruited was small. However, the results of this survey offer novel information on health professionals LBP management and smartphone app recommendation. To the best of our knowledge, this is the first study to investigate health professionals’ recommendation of smartphone apps for the self-management of LBP and provides important knowledge in this area. Future research should explore entry-level training and professional development opportunities for health professionals in digital health, smartphone apps and self-management of LBP. There is also scope for the development of a user-friendly app quality rating tool to guide health professional decision making when recommending apps in clinical practice.

Few health professionals were able to correctly identify LBP guideline self-management recommendations. Greater knowledge and use of guideline-based recommendations to facilitate LBP self-management, particularly in return-to-work facilitation, is required. LBP self-management apps are cost effective, scalable, and accessible and can be used as an adjunct to current modes of LBP management but are underutilized. Apps that were recommended were not specific for LBP self-management. Reasons for not recommending apps included lack of knowledge of app effectiveness and assumptions about digital literacy level of clients. However, those that did recommend apps were confident in choosing and recommending apps and assessing LBP app quality and placed importance on self-management features.

Providing self-management options in LBP is essential to improving outcomes (Foster *et al.*, 2018). Management options are required to decrease health inequality, increase accessibility to health management guidance, and address the economic and personal burden of LBP. Apps offer a novel self-management option that may address these. Health professionals are in an optimal position to recommend apps, and their opinions and recommendations are valued by consumers (Accenture, 2020; Deloitte, 2018). To assist in this process, a user-friendly app assessment tool can assist both consumers and health professionals in appropriate app choice to guide implementation and adoption of app use into LBP management. Easy and appropriate app choice may address the burden of LBP using a scalable, cost-effective, easily accessible, self-management option.

Consumers sought advice from health professionals about apps, highlighting the need for an easy-to-use app assessment method that can assist health professionals and guide consumers in choosing high quality LBP-specific apps from those currently available. This could reduce barriers to recommendation, encourage uptake, and reduce the personal and economic burden of LBP. Promoting digital health tools, such as apps, for self-care may improve self-management and self-efficacy.
